# Characterizing patient-oriented tools that could be packaged with guidelines to promote self-management and guideline adoption: a meta-review

**DOI:** 10.1186/s13012-016-0419-1

**Published:** 2016-04-14

**Authors:** Robin W. M. Vernooij, Melina Willson, Anna R. Gagliardi, the members of the Guidelines International Network Implementation Working Group

**Affiliations:** 1 Iberoamerican Cochrane Centre, Biomedical Research Institute Sant Pau (IIB Sant Pau), Barcelona, Spain; 2 Systematic Reviews and Health Technology Assessments, NHMRC Clinical Trials Centre, University of Sydney, Sydney, Australia; 3 Toronto General Hospital Research Institute, University Health Network, Toronto, Canada

**Keywords:** Clinical practice guidelines, Implementation, Patient engagement, Self-management, Systematic review

## Abstract

**Background:**

Self-management is an important component of care for patients or consumers (henceforth termed patients) with chronic conditions. Research shows that patients view guidelines as potential sources of self-management support. However, few guidelines provide such support. The primary purpose of this study was to characterize effective types of self-management interventions that could be packaged as resources in (i.e., appendices) or with guidelines (i.e., accompanying products).

**Methods:**

We conducted a meta-review of systematic reviews that evaluated self-management interventions. MEDLINE, EMBASE, and the Cochrane Library were searched from 2005 to 2014 for English language systematic reviews. Data were extracted on study characteristics, intervention (content, delivery, duration, personnel, single or multifaceted), and outcomes. Interventions were characterized by the type of component for different domains (inform, activate, collaborate). Summary statistics were used to report the characteristics, frequency, and impact of the types of self-management components. A Measurement Tool to Assess Systematic Reviews (AMSTAR) was used to assess the methodological quality of included reviews.

**Results:**

Seventy-seven studies were included (14 low, 44 moderate, 18 high risk of bias). Reviews addressed numerous clinical topics, most frequently diabetes (23, 30 %). Fifty-four focused on single (38 educational, 16 self-directed) and 21 on multifaceted interventions. Support for collaboration with providers was the least frequently used form of self-management. Most conditions featured multiple types of self-management components. The most frequently occurring type of self-management component across all studies was lifestyle advice (72 %), followed by psychological strategies (69 %), and information about the condition (49 %). In most reviews, the intervention both informed and activated patients (57, 76 %). Among the reviews that achieved positive results, 83 % of interventions involved activation alone, 94 % in combination with information, and 95 % in combination with information and collaboration. No trends in the characteristics and impact of self-management by condition were observed.

**Conclusions:**

This study revealed numerous opportunities for enhancing guidelines with resources for both patients and providers to support self-management. This includes single resources that provide information and/or prompt activation. Further research is needed to more firmly establish the statistical association between the characteristics of self-management support and outcomes; and to and optimize the design of self-management resources that are included in or with guidelines, in particular, resources that prompt collaboration with providers.

**Electronic supplementary material:**

The online version of this article (doi:10.1186/s13012-016-0419-1) contains supplementary material, which is available to authorized users.

## Background

Governments, professional societies, foundations, academic groups, and other organizations worldwide develop guidelines which translate scientific research findings into recommendations that have the potential to enhance health care quality and outcomes [1]. Guidelines are viewed as a foundation for health care planning, delivery, evaluation, and quality improvement [2]. However, the inconsistent use of guidelines has been referred to as a crisis, leading to heterogeneity in clinical practice, over- or underuse of beneficial therapies, preventable harm, suboptimal patient outcomes or experiences, and inefficient use of resources [3, 4]. Efforts to tailor a wide variety of guideline implementation strategies have achieved positive yet modest results. Thus further research is required to understand how guideline implementation can be optimized [5, 6].

Interviews with health care professionals revealed that they struggled to interpret and apply guidelines, and that they desired tools that would facilitate guideline implementation [7]. Systematic reviews show that implementation tools such as guideline summaries, algorithms, point-of-care checklists, and health status reminders enhanced compliance with guideline recommendations [8–10]. Experts have also advocated for the development of guideline-based implementation tools that could be used by patients and providers to clarify the goals of care, understand the underlying evidence, assess the risks and benefits of treatment options, and enable informed decision-making [3, 4]. In other research, we revealed that few guidelines published between 1980 and 2013 offered information or tools to support implementation [11]. We also learned that organizations generating guidelines desired guidance for developing implementation tools [12] given that current guideline development instructional manuals lacked such instructions [13, 14]. We then engaged the international guideline community to produce a framework and considerations with which to assess and adapt existing or develop new guideline implementation tools [15, 16].

Historically, health care professionals were considered to be the primary users of guidelines. Now, it is well-recognized that patients or consumers (henceforth referred to collectively as patients) should be involved in guideline development and are key users of guidelines [17–19]. An emphasis on patient-centered care has also prompted the development of resources to engage patients in their own health care [20–22], and research has optimized the format and content of evidence summaries for the public [23]. So far, little research has examined how to implement patient-oriented tools such as lay language summaries or decision aids into practice [24], and only one study investigated how guidelines can be used as a vehicle for disseminating such patient-oriented implementation tools [25]. In the previous research, we analyzed the content of guidelines and found that 50 % provided information to educate or engage patients [26]. Of these, five guidelines provided information to help clinicians discuss relevant issues with patients, two included information sheets for patients, and seven provided contact information (phone number or web site) where information for patients could be obtained. A systematic review found that patients and members of the public viewed guidelines as sources of health information, support for informed decision-making, and resources to manage their own care but were uncertain about how to find, assess, and use guidelines [27]. This may highlight an opportunity for guideline developers to enhance the implementability of their guidelines by including patient-oriented tools and for providers to share guidelines and associated patient-oriented tools with patients at the point of care. This may improve the adoption and impact of guidelines.

The prevalence of chronic disease is increasing worldwide, and the challenge of addressing the needs of patients with one or more chronic conditions is well-recognized [28]. Self-management has been commonly defined as “the tasks that individuals must undertake to live with one or more chronic conditions” and self-management support as “the systematic provision of education and supportive interventions by health care staff to increase patients’ skills and confidence in managing their health problems, including regular assessment of progress and problems, goal setting, and problem-solving support” [29]. It is distinguished from shared decision-making which involves deliberation between patients and providers about the risks and benefits of health care options to arrive at a mutually agreeable decision about the best course of action for that patient [29]. Self-management is viewed as a complementary to medical care—it is an ongoing process that involves (1) interaction with health care professionals to (2) provide patients with information and education about their condition, its clinical management, options for self-care and what to expect, and activities traditionally provided by health care professionals and, (3) above and beyond that, equip patients with strategies and tools to help them implement and sustain behaviors to cope with their condition and optimize quality of life [30]. A meta-review of self-management support by Taylor et al. found that program components ranged along a spectrum from simple summaries of disease-specific information to multifaceted interventions [31]. However, the review did not identify one or more components as crucial [31]. Effectiveness was variable across interventions and conditions. For example, action plans were beneficial for asthma management while self-monitoring was beneficial for hypertension management. Educational information was a core component of all self-management interventions evaluated in 102 systematic reviews, including 969 randomized controlled trials (RCTs). Qualitative studies included in the meta-review revealed that effectiveness may be associated with good communication with providers at the point of care about self-management.

An increasing number of patients with chronic conditions may require and benefit from self-management, and self-management may be an intervention best introduced by providers at the point of care. It may be possible for guidelines to provide patients and providers with resources that enable the interactive, educational, and supportive domains of self-management. First, we need to understand the components of self-management interventions that are both effective and of a nature such that they could be included as resources in print or electronic versions of guidelines. The primary purpose of this study was to systematically review published research on self-management to describe the self-management components that have been evaluated as effective and could potentially be packaged in (i.e., as appendices) or with guidelines (i.e., as accompanying products) for use by patients and physicians to promote discussions about and the uptake of self-management. A secondary purpose was to assess the effectiveness of single versus multifaceted types of self-management interventions, as this has implications for the resources required to develop and include one or more self-management components in or with guidelines. Ultimately, the inclusion of such resources in guidelines may improve the implementation and integration of self-management into care delivery and self-care and, by enhancing the implementability of guidelines, lead to greater adoption of guidelines and associated beneficial outcomes.

## Methods

### Approach

Given the large number of published systematic reviews on self-management, a meta-review of systematic reviews on the impact of self-management programs was conducted using standard systematic review methods [32]. The Preferred Reporting Items for Systematic Reviews and Meta-Analyses (PRISMA) criteria guided the conduct and reporting of the review [33]. Data were publicly available so institutional review board approval was not necessary. A protocol for this review was not registered. Data extracted from eligible systematic reviews describing self-management interventions were thematically analyzed to categorize them according to type of self-management components and assess observable patterns of association between type of self-management components and beneficial outcomes reported by eligible studies.

### Scoping

To plan for the full-scale review, a preliminary scan of relevant literature was undertaken by assessing the nature of eligible studies included in the Taylor et al. meta-review [31]. This refined the objectives of the review, leading to the development of a population, intervention, comparisons, and outcomes (PICO) statement and contributed to the development of search parameters and screening criteria. The *Population* of interest was adult patients or consumers aged 18 years and older with any chronic health condition including communicable and non-communicable diseases with no definite cure. The *Intervention* of interest was self-management interventions delivered in any format, potentially referred to instead as self-care, self-monitoring, or self-help interventions. Self-management interventions were comprised at minimum of an informational or educational component and potentially additional supportive (behavioral, educational, psychological, clinical) components to encourage people to take an active role in their own health and better manage the condition itself and their overall well-being [31]. Relevant study *Comparisons* included patients with and without exposure to self-management interventions, or before or after exposure to self-management interventions, or patients receiving different types of self-management interventions, or comparison of any type of self-management intervention with any type of alternative intervention that was meant to address the same health condition issues as the self-management intervention. Self-management interventions are usually targeted at maintaining or improving life with the condition, rather than improving the condition itself; therefore, *Outcomes* of interest included any functional outcomes that were reported by the study, including but not limited to adherence to medical care, physiological function, overall well-being, return to daily living, pain, social or psychological factors, or adoption of new activities or behaviors, measured clinically, or with instruments, questionnaires, or interviews.

### Searching

MEDLINE, EMBASE, the Cochrane Library, and CINAHL were searched in February 2015 for studies published from January 1, 2005 to that date which evaluated self-management interventions. A 10-year time span was used to capture the components of self-management interventions developed more recently, thereby enabling a current-day description of informational or educational self-management components that could accompany guidelines. To ensure that our meta-review was comprehensive, we also applied our screening criteria to review the 102 systematic reviews included in the Taylor et al. meta-review [31] which included systematic reviews of RCTs published from January 1993 to June 2012 (as reported by authors). Our meta-review updates and expands on the meta-review conducted by Taylor et al. [31] by including nearly two additional years of published systematic reviews and systematic reviews that may have included study designs other than RCTs. The search strategy was purposefully broad to be as inclusive as possible and included both Medical Subject Headings and keywords (Additional file 1). Searches were limited to English language to avoid the cost of translation and expedite completion of the review.

### Screening

Titles and abstracts of search results were reviewed independently by ARG and a research assistant following a pilot test during which they independently screened the first 100 results, then compared and discussed their selections to establish a shared understanding of the screening criteria. All items selected by at least one reviewer were retrieved for further assessment. If more than one publication described a single study, the most recent or complete publication was included. Selection criteria included systematic reviews or meta-analyses that described the impact of self-management interventions meeting the criteria specified in the abovementioned PICO statement. As search results were reviewed, selection criteria were expanded to specify studies that were not eligible. Studies were excluded if they focused on (1) clinical treatment; (2) individuals that did not have a chronic condition but temporarily required self-monitoring, for example, as part of rehabilitation after surgery or an accident; (3) contextual or environmental conditions that may influence or moderate the impact of self-management, for example, the role of family; (4) information-seeking patterns among patients or the well public; (5) promotion of self-care for the elderly without any underlying disease or as a means of preventing a disease; (6) self-management interventions with no educational or informational component, for example, exercise only; or (7) barriers to self-management. Guidelines as a publication type, protocols, proceedings, abstracts, letters, editorials, or commentaries were not eligible.

### Data extraction

A data extraction form was developed to collect information on study design (number/type of eligible studies), disease or condition, number and type of participants, methodological assessment, intervention details (content, delivery mode, frequency and duration, audience, personnel), and findings. ARG and a research assistant pilot-tested the form on three articles through four iterations until data extraction was consistent. The research assistant proceeded to extract data from all eligible studies. Extracted data were confirmed independently by RWMV and MW who reviewed each study and edited or added to the extracted data.

### Quality assessment

The methodological quality of eligible studies was assessed with A Measurement Tool to Assess Systematic Reviews (AMSTAR), for which a score of 0 to 4, 5 to 8, and 9 to 11 represent low, moderate, and high quality, respectively [34]. ARG and the research assistant independently assessed 10 studies, then compared and discussed their findings to achieve a shared understanding of how to apply the criteria, following which the research assistant assessed remaining studies.

### Data analysis

Extracted data were summarized to describe the characteristics of eligible studies including years when published, country of first author, the number of included studies and participants, and the quality of included studies if assessed. Interventions were categorized as educational sessions (single or multiple provider and/or lay leader sessions during which information was conveyed to individuals or groups in-person or by virtual means), self-directed guides (print material, computer, Internet, or information technology if more than one electronic delivery mechanism was used), or counseling (brief in-person or virtual interaction during which providers and/or lay leaders provided patients with recommendations, reminders or encouragement). Data were not pooled due to heterogeneity in the study design, nature of interventions, and reported outcomes.

The primary purpose of this review was to describe the components of self-management interventions. To do so, we used a modified version of the taxonomy of self-management components generated by the Taylor et al. meta-review in which we eliminated items pertaining to clinical care or the provision of equipment and retained items that could potentially be included in or with guidelines [31]. The modified taxonomy of components were categorized into three domains adopted from work by Grande et al. [20] corresponding to the interactive, educational, and supportive aspects of self-management: *informing*—providing knowledge about their condition and an understanding of how to manage it; *activating*—providing information or tools to prompt action for actively managing the condition and enhancing quality of life; and *collaborating*—providing information or linkages that lead to interaction with health providers or agencies. Table 1 shows the resulting taxonomy used to describe the components of self-management interventions and provides examples of each. Two authors (RWMV, ARG) independently used thematic analysis to peruse the details of self-management interventions extracted from included studies and categorize them as one or more components. They compared findings and discussed discrepancies to achieve consensus.

**Table 1:** Taxonomy of self-management components

Domain^a^	Component^b^	Examples
Inform Information that provides patients with knowledge about their condition and an understanding of how to manage it	Condition	Information and evidence about the condition, prognosis, what to expect, and its management
	Activities of daily living	Information and advice on how to undertake generic activities such as hygiene, dressing, preparing meals, transportation
	Lifestyle advice	Information and guidance on lifestyle behaviours that support disease management
Activate Information or tools to prompt action for actively managing the condition and enhancing quality of life	Support for condition	Reminders, diaries, or other prompts to support adherence to medication or recommended lifestyle behaviours
	Action plans for condition	Guidance specific to medical condition, providing signs of worsening condition, how to self-adjust treatment, and response if deterioration continues
	Monitoring	Self-evaluation tools to log and monitor physiological measures for personal assessment and to share with clinicians
	Psychological strategies	Mechanisms for problem-solving, goal-setting, reframing, relaxation
Collaborate Information or mechanisms that lead to interaction and engagement	Communication with clinicians	Guidance and prompts to facilitate communication with health care professionals
	Available resources	Links to or contact details for organizations that offer information, psycho-social support, or financial aid
	Social support	Links to or contact details for organizations that offer support, mentoring, or socializing

^a^From Grande et al. [20]

^b^Adapted from Taylor et al. [31]: patient medical care and equipment were removed from the original framework as these cannot be packaged in or with guidelines

The secondary purpose of this review was to assess whether single-component self-management interventions were as effective as multifaceted self-management interventions. Eligible studies were categorized by self-management component (Table 1) and by format of delivery as single- (one of education session, self-directed guide, or counseling) or multifaceted (more than one of these). Given the diversity of conditions, interventions employed, and outcomes reported, we did could not pool and statistically analyze the impact of interventions. Instead, to explore potential trends, outcomes as reported by the authors were categorized as all positive (improved outcomes), all negative (no change or worsening outcomes), or mixed (a blend of improved, no change, or worsening outcomes).

## Results

### Search results

**Fig. 1:**
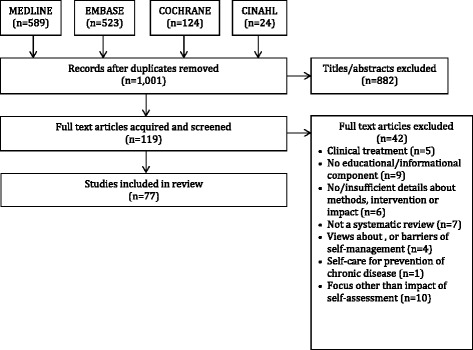
PRISMA diagram of search results

A total of 1001 unique articles were identified by search strategies across all sources of which 882 were excluded based on screening of titles and abstracts, leaving 119 full-text articles to be screened. Of these, 42 were excluded due to study design (7) or lack of details about methods, intervention, or impact (6); focused on prevention (1) or clinical treatment (6), views about, or barriers of self-management (4) did not evaluate the impact of self-management (10) or were based on an intervention that did not include an educational or informational component (9). As a result, 77 studies were included in the review (Fig. 1). Data extracted from each review appear in Additional file 2 [35–111].

### Characteristics of eligible studies

Of the 77 eligible reviews, 19 (25.0 %) were conducted in the UK, 18 (23.0 %) in the USA, 10 (13.0 %) in Australia, and 5 (6.5 %) in Canada. Other reviews conducted in Europe (other than the UK) emerged from the Netherlands, Switzerland, and Denmark. The majority of reviews were conducted in the year 2010 or afterwards (70.0 %). The largest proportion of reviews by year published occurred in 2013 (16.9 %). The mean number of studies included in eligible reviews was 23.6 (range 2.0 to 163.0). Eligible reviews addressed a wide range of clinical topics that were categorized as metabolic conditions which were all related to diabetes (23, 29.9 %); musculoskeletal conditions such as arthritis and back pain (12, 15.6 %); reviews of a variety of chronic conditions (12, 15.6 %); cardiovascular conditions such as angina, hypertension, heart disease, and stroke (11, 14.3 %); pulmonary conditions such as asthma and chronic obstructive pulmonary disease (7, 9.1 %); other conditions such as cancer pain, irritable bowel syndrome, epilepsy, multiple sclerosis, and kidney disease (7, 9.1 %); and mental illness including anxiety and depression (5, 6.5 %).

### Quality assessment of eligible studies

Quality assessment found that 14, 44, and 18 studies had a low, moderate, and high risk of bias (Additional file 2).

### Characteristics of self-management

Table 2 summarizes the characteristics of self-management interventions reported in 75 of 77 eligible reviews that provided sufficient detail to categorize them according to domain, component, and delivery format. Of these, 54 reviews included studies that largely employed single types of interventions (delivery format). These were educational sessions in 38 studies, self-directed guides in 16 reviews of which 14 involved self-directed guides in electronic form (computer or phone application, Internet), one review in print format, and one review in which both electronic and print self-directed guides were offered. A further 21 reviews included studies that offered a variety of single interventions or multifaceted interventions. These included 8 reviews of education, self-directed guides, and counseling; 7 reviews of education and self-directed guides; 4 reviews of education and counseling; and 2 reviews of self-directed guides and counseling.

**Table 2:** Summary of self-management characteristics and outcomes reported in included studies

Self-management domain	Intervention outcomes by delivery format (number of studies)^a^									Sub-total
	Education *n*=38			Self-directed *n*=16			Multifaceted *n*=21			
	+	±	−	+	±	−	+	±	−	
Inform	1	1	0	2	1	0	0	0	0	5
Activate	2	1	1	5	1	1	1	0	0	12
Collaborate	0	0	0	0	0	0	0	0	0	0
Inform and activate	14	8	2	1	0	0	9	2	0	36
Activate and collaborate	1	0	0	0	1	0	0	0	0	2
Inform, activate and collaborate	6	1	1	2	2	0	4	5	0	21
Sub-total	24	11	4	10	5	1	15	7	0	75

*+* all reported results are positive (improved outcomes), *−* all reported results are negative (no change or worsening outcomes), *±* mixed results (a blend of improved, no change, or worsening outcomes)

^a^The total is 75 because two studies did not provide sufficient detail to categorize the type of self-management support offered; one used education and achieved a positive result; one used education, self-directed guides, and counseling and achieved a positive result [62, 85]

The majority of reviews included self-management interventions that involved multiple domains (i.e., inform, activate, collaborate) and components. For example, 36 reviews included studies with interventions that provided components to both inform and activate patients, while 21 reviews included studies with interventions that provided components to inform and activate patients and promote collaboration.

### Impact of self-management

Table 2 summarizes the reported outcomes of eligible reviews. The majority of reviews reported positive results for all measures reported (47/75, 62.7 %). This included 23 reviews of educational sessions, 10 reviews of self-directed guides, and 14 reviews of multifaceted interventions. Mixed results were reported in 23 reviews (11 education, 5 self-directed, 7 multifaceted), and 5 reviews (4 education, 1 self-directed) failed to show the impact of self-management on reported outcomes.

When outcomes were perused by the type of self-management domain (i.e., inform, activate, collaborate), it appeared that interventions which activated patients may have been associated positive impact. This may have been supplemented by interventions that also informed patients. For example, positive results were achieved in 58.3 % (7/12) of interventions based on activation alone, 66.7 % (24/36) in combination with information, and 57.1 % (12/21) in combination with information and collaboration. If reviews that achieved both positive and mixed results were considered, then 83.3 % (10/12) of interventions based on activation alone, 94.4 % (34/36) in combination with information, and 95.2 % (20/21) in combination with information and collaboration were successful. This apparent trend was observed across educational, self-directed, and multifaceted interventions. Positive results were also achieved in two reviews that evaluated interventions that stimulated both activation and collaboration. Of the 4 studies that did not include activation (2 educational, 2 self-directed interventions), 3 achieved positive and 1 achieved mixed results.

### Frequency and impact of self-management by condition

Table 3 summarizes the frequency of self-management interventions by domain and component for different types of conditions.

**Table 3:** Self-management characteristics featured in reviews by condition

Self-management characteristics		Featured in reviews (*n*, *n*/75)^a^	Featured in reviews by condition (*n*, *n*/75)^a^						
Domain	Component		Metabolic	Mental illness	Cardiovascular	Musculoskeletal	Pulmonary	Variety of chronic diseases	Other
Inform	Condition	37	12	1	5	5	3	4	7
		49.3	16.0	1.3	6.7	6.7	4.0	5.3	9.3
	Activities of daily living	9	4	1	0	2	0	0	2
		12.0	5.3	1.3	0.0	2.7	0.0	0.0	2.7
	Lifestyle advice	54	18	1	6	9	5	8	7
		72.0	24.0	1.3	8.0	12.0	6.7	10.7	9.3
Activate	Support for condition	26	9	0	2	7	3	1	4
		34.7	12.0	0.0	2.7	9.3	4.0	1.3	5.3
	Action plans for condition	30	8	0	4	6	6	2	4
		40.0	10.7	0.0	5.3	8.0	8.0	2.7	5.3
	Monitoring	31	13	0	5	3	3	3	4
		41.3	10.7	0.0	6.7	4.0	4.0	4.0	5.3
	Psychological strategies	52	14	5	4	8	5	8	8
		69.3	18.7	6.7	5.3	10.7	6.7	10.7	10.7
Collaborate	Communicate with clinicians	9	1	0	1	2	0	2	3
		12.0	1.3	0.0	1.3	2.7	0.0	2.7	4.0
	Link with resources	2	0	0	0	0	1	0	1
		2.7	0.0	0.0	0.0	0.0	1.3	0.0	1.3
	Social support	14	4	1	0	2	0	4	3
		18.7	5.3	1.3	0.0	2.7	0.0	5.3	4.0

^a^The total is 75 because two studies did not provide sufficient detail to categorize the type of self-management support offered; one used education and achieved a positive result; one used education, self-directed guides, and counseling and achieved a positive result [62, 85]

When examined by domain, self-management components that promoted collaboration with health care professionals or others was the least frequently used form of self-management across all studies. The most frequently occurring type of self-management components across all included studies was lifestyle advice (54, 72.0 %), followed by psychological strategies (52, 69.3 %) and information about the condition (37, 49.3 %). The least frequent types of self-management components were links to supportive care resources (2, 2.7 %), information about accomplishing the activities of daily living (9, 12.0 %), and guidance or prompts to facilitate communication with health care professionals (9, 12.0 %). When examined by condition, the frequency of the types of self-management components differed. For example, among the reviews of mental illness, psychological strategies were the most common. Among the reviews of pulmonary conditions, action plans were the most frequently used form of self-management component, followed closely by psychological strategies and lifestyle advice.

Most conditions featured multiple types of self-management components. There did not appear to be trends in the characteristics or impact of self-management by condition. For example, metabolic disease (all diabetes) was the focus of the greatest proportion of reviews (23, 29.9 %). Among these, 13, 3, and 6 were based on educational, self-directed, and multifaceted interventions, respectively (Additional file 2). In 13 reviews that focused on educational interventions by activation alone (1), information and activation (8) or information, activation and collaboration (3), 83.3 % achieved positive or mixed results. In 3 reviews that focused on self-directed interventions by activation alone (1), information and activation (1), or information, activation, and collaboration (1), all achieved positive or mixed results. In 6 reviews that focused on multifaceted interventions by activation alone (1), information and activation (3) or information, activation and collaboration (2), all achieved positive or mixed results.

## Discussion

This review was conducted to identify and describe the characteristics of effective self-management interventions that could be packaged in or with guidelines as a means of delivering them directly to patients or indirectly through their providers. Educational sessions were the most frequently used format for delivering self-management, followed by self-directed guides which were largely electronically available. Interventions were based on multiple self-management domains and components, most often by offering information about recommended lifestyle choices and by activating patients to adopt and maintain those lifestyle choices through psychological strategies. It appeared that single or multifaceted interventions were associated with positive outcomes. This included informational-only self-management components and self-management components that included activation alone or in combination with other types of support. Activation was most frequently impactful when combined with informational support.

Several implications can be drawn from these findings. The modified taxonomy of self-management used in this study was easy to apply and able to characterize all of the intervention components described in the included systematic reviews [31]. Therefore, it was further validated and can be used by guideline developers and others as the basis for planning and developing patient-oriented guideline implementation tools that support self-management. Most conditions employed multiple types of self-management components; however, it appeared that even single self-management interventions (based on delivery format) can result in beneficial outcomes. The majority of included studies offered evidence that resources which inform or activate patients can achieve beneficial outcomes. This is relevant to guideline developers who often possess few resources with which to develop and implement guidelines and must therefore decide how to allocate scarce resources by prioritizing the strategies they will use for guideline implementation [12]. This finding is similar to that of a recent meta-review of 25 systematic reviews that compared direct and indirect effect size and dose-response of single and multifaceted strategies which showed no benefit of multifaceted over single strategies [112].

Although studies were few, and the association between self-management characteristics and various outcomes remains to be established, there appears to be multiple types of self-management components that may be effective and could be packaged in or with guidelines. This includes information about the condition and its management, how to accomplish activities of daily living, and lifestyle behaviors that support disease management (inform); tools that enable disease and lifestyle management such as diaries, action plans, monitoring measures and logging templates, and tips for problem-solving, goal-setting, or relaxation (activate); and prompts for when and how to communicate with health care providers or other supportive care agencies (collaborate). Given that, in other research, patients favorably viewed guidelines as a useful source of self-management support [27], and our research showed that few guidelines offered such resources [11], this study reveals numerous opportunities to enhance guidelines as a means of promoting the adoption of guidelines and self-management. Ultimately, the optimal use of guidelines and enhanced integration of self-management into care delivery and self-care may improve the health status of patients with chronic conditions.

Other researchers have also highlighted the need to better implement tools that support patient engagement in their own health care [25] and that guidelines offer a potential vehicle for doing so [26]. Although, in previous research, we produced a framework and considerations with which to assess and adapt existing, or develop new guideline implementation tools [15, 16], further research is needed to apply this guidance to the development of informational or activating tools that specifically support self-management. A variety of theories offer insight on how patient perspectives influence their health-related views and behavior, and can be used to design and then evaluate self-management guideline tools. These include the Health Belief Model and Theory of Planned Behavior [113, 114]. Normalization Process Theory provides insight on how sociological processes influence the implementation of innovations and could be used to examine how patients and providers adopt and integrate self-management [115]. The PRECEDE-PROCEED model is another useful framework by which to plan or evaluate self-management tools based on patient-specific and external factors that may influence their impact [116].

While patients may see the benefit of accessing self-management support through guidelines, research shows that many patients are not aware of guidelines or where to find them [11]. The Taylor et al. meta-review included a complementary analysis of 30 qualitative systematic reviews and 61 studies on the implementation of self-management programs which revealed that interventions such as education and training, feedback, prompts or reminders, equipment, and financial incentives were needed so that providers offered self-management support to their patients [31]. Other systematic reviews similarly found that educational strategies were needed to prompt health care professionals to adopt patient-centered approaches in clinical consultations such as shared decision-making [117, 118]. The qualitative review included in the Taylor et al. meta-review also emphasized that the effectiveness of self-management was enhanced through good collaboration with providers at the point of care. This review revealed that self-management support to promote collaboration with health care professionals or others was the least frequently used form of self-management across all studies. Thus further research is needed to develop and evaluate resources such as question prompt lists for patients about when and how to communicate with health care providers [119]. Further research is also needed to investigate whether and how guidelines prompt health care professionals to engage in conversations with patients.

A variety of factors limit the interpretation and application of these findings. The literature search, which was not peer-reviewed by another librarian, and screening process may not have identified all relevant studies although we employed a comprehensive search strategy and multiple independent screeners selected eligible articles. The characteristics of self-management interventions in eligible studies may not have been accurately categorized even though data were independently extracted by multiple authors and a research assistant. In part, this is due to the fact that self-management interventions were not well-described in all eligible reviews and, even if the intervention was stated, the type of self-management domain and component was not always explicit and had to be inferred. This may also be due to a general lack of consensus on the definition of self-management and a corresponding confusing array of interventions labelled as self-management in the literature, as was observed here and by Taylor et al. [31]. Others have distinguished self-management education (informs decision-making, self-care, problem-solving) from self-management support (enables adoption and maintenance of self-management behaviors that influence functional outcomes), yet this was not clear in many of the included studies [120, 121]. Moreover, of the 77 studies included, 44 and 18 were found to have a moderate and high risk of bias, respectively. Thus the results reported by 80.5 % of the included reviews should be interpreted with caution and influence the overall findings reported here. That being said, the modified Taylor et al. taxonomy proved to be a useful means of distinguishing between the components of self-management interventions [31]. This systematic review was exploratory in nature and revealed a potential association between the type of self-management support that could be included in or with guidelines and positive impact. However, given the diversity of conditions, interventions employed, and outcomes reported, we were not able to generate a statistical measure of association. Future research could repeat this work but focus on RCTs evaluating single diseases in an effort to examine effect size and confidence intervals, and pool that data. The underlying mechanism of self-management interventions associated with impact could be examined if such a review used realist approach [122]. In that work, self-management interventions could be categorized based on outcome quadrants suggested by De Silva et al. knowledge, technical skill, self-efficacy, and behavior change [123]. Future research could also examine the content of guidelines to characterize the self-management support they offer, thereby identifying exemplars that others could emulate, and gaps where self-management support may be needed.

## Conclusions

In this meta-review, single or multifaceted educational or self-directed self-management interventions that included activation support (i.e., reminders, diaries, action plans, tools to monitor health status, psychological strategies for problem-solving) may have reinforced general information about the condition and lifestyle advice, and contributed to the positive impact of self-management interventions as reported in included studies. Given that, in previous research, patients desired self-management support and few guidelines offered such tools, this study revealed numerous opportunities by which to enhance guidelines in a way that supports self-management to contribute to the improved health of patients with a variety of chronic conditions. Further research is needed to establish the statistical association between the characteristics of self-management support and outcomes and to optimize the design of self-management tools for both health care providers and patients that are included in or with guidelines.

## Additional files

Additional file 1: MEDLINE search strategy. Search strategy as applied in MEDLINE. (DOCX 13.2 kb)

Additional file 2: Data extracted from eligible studies. Data extracted from eligible studies. (DOCX 25.5 kb)
