# Supplementary material for: Characterizing patient-oriented tools that could be packaged with guidelines to promote self-management and guideline adoption: a meta-review
Source: Implement Sci. 2016 Apr 14;11:52. doi: 10.1186/s13012-016-0419-1 (PMC4832541; doi:10.1186/s13012-016-0419-1)
Supplement: Supplementary file 1 — MEDLINE search strategy. Search strategy as applied in MEDLINE. (DOCX 13.2 kb) [file 13012_2016_419_MOESM1_ESM.docx]

Additional File 1. MEDLINE search strategy

--------------------------------------------------------------------------------

1 Self Care/ (18516)

2 (self-management or self management).mp. (7127)

3 1 or 2 (21433)

4 limit 3 to (english language and yr="2005 -Current" and "all adult (19 plus years)") (7816)

5 limit 4 to "reviews (best balance of sensitivity and specificity)" (455)

6 limit 3 to (english language and yr="2005 -Current" and "all adult (19 plus years)") (7816)

7 limit 6 to (meta analysis or systematic reviews) (300)

8 5 or 7 (589)

***************************
