# Supplementary material for: Characterizing patient-oriented tools that could be packaged with guidelines to promote self-management and guideline adoption: a meta-review
Source: Implement Sci. 2016 Apr 14;11:52. doi: 10.1186/s13012-016-0419-1 (PMC4832541; doi:10.1186/s13012-016-0419-1)
Supplement: Supplementary file 2 — Data extracted from eligible studies. Data extracted from eligible studies. (DOCX 25.5 kb) [file 13012_2016_419_MOESM2_ESM.docx]

Additional File 2. Data extracted from eligible articles

| Study  Risk of bias [34] | Condition | Self-management characteristics | | | Outcome |
| --- | --- | --- | --- | --- | --- |
|  |  | Delivery format | | Domain (component) |  |
| Siantz 2014 [35]  moderate | Mental illness | Education session (provider, lay leader) | Inform (condition, activities of daily living, lifestyle advice), activate (psychological strategies), collaborate (social support) | | + |
| Bossen 2014 [36]  moderate | Chronic disease | Self-directed guide (Internet) | Inform (lifestyle advice) | | +/- |
| Kivela 2014 [37]  moderate | Chronic disease | Self-directed guide (information technology) | Activate (psychological strategies) | | + |
| Zhai 2014 [38]  moderate | Diabetes type 2 | Self-directed guide (information technology) | Inform (lifestyle advice), activate (monitoring) | | + |
| Bolen 2014 [39]  moderate | Diabetes type 2 | Education session (provider, Internet) | Inform (lifestyle advice), activate (action plans, monitoring, psychological strategies), collaborate (social support) | | + |
| Attridge 2014 [40]  high | Diabetes type 2 | Education session (provider) | Inform (condition, lifestyle advice), activate (support for condition, psychological strategies) | | + |
| McGillion 2014 [41]  moderate | Chronic stable angina | Education session (provider) | Inform (lifestyle advice), activate (monitoring, psychological strategies) | | + |
| Zwerink 2014 [42]  high | Chronic Obstructive Pulmonary Disease | Education session (provider) | Inform (lifestyle advice), activate (action plan, psychological strategies) | | + |
| Kroon 2014 [43]  high | Osteoarthritis | Education session (provider) | Inform (lifestyle advice), activate (support for condition, action plan, psychological strategies) | | +/- |
| Kopke 2014 [44]  high | Multiple Sclerosis | Education session or self-directed guide (provider, print material) | Inform (lifestyle advice), activate (support for condition) | | + |
| Mayo-Wilson  2013 [45]  high | Anxiety disorders | Self-directed guide (information technology) | Activate (psychological strategies) | | + |
| Burns 2013 [46]  low | Chronic disease | Self-directed guide (Internet) | Inform (condition) | | + |
| Beatty 2013 [47]  moderate | Chronic disease | Self-directed guide (Internet) | Activate (psychological strategies) | | +/- |
| Franek 2013 [48]  moderate | Chronic disease | Education session (provider, lay leader) | Inform (lifestyle advice), activate (support for condition, action plans, psychological strategies) and Collaboration (social support) | | + |
| McDermott 2013 [49]  moderate | Chronic disease | Self-directed guide (computer) | Inform (condition, lifestyle advice) | | + |
| Kuijpers 2013 [50]  moderate | Chronic disease | Self-directed guide (Internet) | Inform (condition, lifestyle advice), activate (monitoring, action plan, psychological strategies) and collaborate (clinician, social support) | | + |
| Stellefson 2013 [51]  moderate | Chronic disease | Self-directed guide (Internet) | Inform (lifestyle), activate (monitoring, psychological strategies), collaborate (clinician, social support) | | + |
| Pal 2013 [52]  high | Diabetes type 2 | Self-directed guide (information technology) | Inform (lifestyle advice), activate (action plans, monitoring), collaborate (social support) | | +/- |
| Torenholt 2013 [53]  low | Diabetes | Education session (provider) | Inform (condition, activities of daily living, lifestyle advice), activate (support for condition, psychological strategies) | | +/- |
| El-Gayar 2013 [54]  moderate | Diabetes | Education session or self-directed guide (information technology) | Inform (lifestyle advice), activation (support for condition, action plans, monitoring) | | + |
| Carter 2013 [55]  low | Diabetes | Education session or self-directed guide (provider, print material, information technology) | Inform (condition, lifestyle advice), activate (support for condition, monitoring, psychological strategies), collaborate (social support) | | + |
| Clarkesmith 2013 [56]  high | Atrial Fibrillation | Education session (provider) | Inform (condition, lifestyle advice), activate (support for condition, action plans) | | - |
| Cumberworth 2013 [57]  low | Heart Disease | Education session (provider) | Activate (monitoring) | | - |
| De Freitas 2013 [58]  moderate | Temporomandibular disorders | Education session (provider) | Inform (condition, activities of daily living, lifestyle advice), activate (action plans) | | +/- |
| Malanda 2012 [59]  high | Diabetes type 2 | Education session (provider) | Activate (monitoring) | | +/- |
| Nam 2012 [60]  moderate | Diabetes type 2 | Education session (provider) | Inform (condition, activities of daily living, lifestyle advice), activate (action plans, monitoring, psychological strategies) | | + |
| Newlin 2012 [61]  low | Diabetes | Education session (provider, lay leader) | Inform (condition, lifestyle advice), activate (support for condition), collaborate (social support) | | +/- |
| Steinbekk 2012 [62]  moderate | Diabetes | Education session (provider) | NR | | + |
| Tshiananga 2012 [63]  moderate | Diabetes | Education session or counseling or self-directed guide (provider, lay leader) | Inform (activities of daily living), activate (psychological strategies), collaborate (clinicians, social support) | | + |
| Forster 2012 [64]  high | Stroke | Education session or self-directed guide (provider, print material, computer) | Inform (condition, lifestyle advice), activate (psychological strategies) | | + |
| Ciere 2012 [65]  moderate | Heart Failure | Self-directed guide (information technology) | Activate (monitoring), collaborate (clinician) | | +/- |
| Bentsen 2012 [66]  low | Chronic Pulmonary Disease | Education session (provider) | Inform (condition, lifestyle advice), activate (action plan, psychological strategies) | | +/- |
| Tan 2012 [67]  moderate | Chronic Obstructive Pulmonary Disease | Education session (provider) | Inform (condition, lifestyle advice), activate (support for condition, action plans, psychological strategies), collaborate (available resources) | | + |
| Wong 2012 [68]  high | Chronic Obstructive Pulmonary Disease | Education session (provider) | Activate (action plan), Collaborate (clinician) | | + |
| Moullec 2012 [69]  moderate | Asthma | Education session (provider) | Inform (condition), activate (support for condition, action plan, monitoring) | | +/- |
| Gross 2012 [70]  high | Neck Pain | Education session or self-directed guide (provider, print material, information technology) | Inform (condition), activate (action plans, monitoring, psychological strategies) | | +/- |
| Oliveira 2012 [71]  moderate | Non-specific Low Back Pain | Education session or self-directed guide (provider, information technology) | Inform (lifestyle advice), activate (support for condition, monitoring) | | + |
| Koller 2012 [72]  low | Cancer Pain | Education session (provider) | Inform (condition, lifestyle advice), activate (monitoring, psychological strategies), Collaborate (clinician) | | + |
| Coull 2011 [73]  moderate | Depression | Self-directed guide (Internet) | Activate (psychological strategies) | | + |
| Cuijpers 2011 [74]  moderate | Depression | Self-directed guide (Internet, print material) | Activate (psychological strategies) | | + |
| Inouye 2011 [75]  low | Chronic disease | Education session (provider) | Inform (condition, lifestyle advice), activate (psychological strategies) | | + |
| Li 2011 [76]  high | Diabetic Kidney Disease | Education session (provider) | Inform (activities of daily living, lifestyle advice), activate (psychological strategies) | | + |
| Lennon 2011 [77]  low | Stroke | Education session (provider) | Inform (condition, lifestyle advice), activate (action plans, psychological strategies) | | +/- |
| Bloomfield 2011 [78]  high | Thromboembolism | Education session (provider) | Inform (condition), activate (support for condition, action plans, monitoring) | | +/- |
| Du 2011 [79]  moderate | Musculoskeletal Pain | Education session or self-directed guide (provider or lay leader, computer) | Inform (condition, lifestyle advice), activate (support for condition, action plans, psychological strategies), collaborate (clinician) | | + |
| Rae-Grant 2011 [80]  Risk of bias: moderate | Multiple Sclerosis | Education session or counseling or self-directed guide (provider, lay leader, information technology) | Inform (condition, activities of daily living, lifestyle advice), activate (psychological strategies), collaborate (clinician, social support) | | + |
| Minet 2010 [81]  moderate | Diabetes type 2 | Education session or counseling (provider) | Inform (condition, lifestyle advice), activate (psychological strategies) | | + |
| Heinrich 2010 [82]  moderate | Diabetes type 2 | Education session (provider) | Inform (condition, lifestyle advice), activate (action plans, monitoring, psychological strategies) | | + |
| Matteson 2010 [83]  low | Chronic kidney disease | Education session (provider) | Inform (condition), activate (action plans, monitoring, psychological strategies) | | + |
| Dorn 2010 [84]  moderate | Irritable Bowel Syndrome | Education session or counseling or self-directed guide (provider, print material) | Inform (condition, lifestyle advice), activate (action plans, psychological strategies) | | + |
| Bray 2010 [85]  moderate | Hypertension | Education or counselling or self-directed guide (provider, Internet, print material) | NR | | + |
| Saksena 2010 [86]  low | Hypertension | Self-directed guide (information technology) | Inform (condition, lifestyle advice), activate (psychological strategies), collaborate (clinician) | | +/- |
| Albano 2010 [87]  moderate | Arthritis | Education session or counseling or self-directed guide (provider, print material, information technology) | Inform (condition, activities of daily living, lifestyle advice), activate (support for condition, action plans, monitoring, psychological strategies), collaborate (clinician, social support) | | +/- |
| Iversen 2010 [88]  low | Arthritis | Education session or counseling or self-directed guide (provider, print material) | Inform (lifestyle advice), activate (support for condition, action plan, psychological strategies), collaborate (social support) | | +/- |
| Graziano 2009 [89]  moderate | Diabetes type 2 | Education session or counseling (provider, information technology) | Inform (lifestyle advice), activate (action plans, monitoring, monitoring) | | + |
| Duke 2009 [90]  high | Diabetes type 2 | Education session (provider) | Inform (condition, lifestyle advice), activate (support for condition, action plans, monitoring, psychological strategies) | | - |
| Hwang 2009 [91]  low | Chronic Heart Failure | Education session (provider) | Inform (lifestyle advice) | | + |
| Williams 2008 [92]  moderate | Chronic disease | Self-directed guide (computer) | Activate (psychological strategies) | | - |
| Conn 2008 [93]  moderate | Chronic disease | Education session or counseling or self-directed guide (provider, print material) | Inform (lifestyle advice), activate (monitoring, psychological strategies), collaborate (social support) | | +/- |
| Khunti 2008 [94]  moderate | Diabetes type 2 | Education session (provider) | Inform (condition, lifestyle advice), activate (support for condition, monitoring), collaborate (clinician) | | - |
| Mason 2008 [95]  moderate | Chronic kidney disease | Education session (provider) | Inform (condition, lifestyle advice), activate (support for condition, action plans, monitoring, psychological strategies), collaborate (clinician, social support) | | + |
| Brox 2008 [96]  moderate | Low Back Pain | Education session (provider) | Inform (lifestyle advice), activate (support for condition, psychological strategies) | | + |
| Bradley 2008 [97]  moderate | Epilepsy | Education session or counseling or self-directed guide (provider, print material) | Inform (condition, lifestyle advice), activate (support for condition, psychological strategies), collaborate (available resources, social support) | | +/- |
| Foster 2007 [98]  high | Chronic disease | Education session (provider) | Inform (lifestyle advice) | | +/- |
| Conn 2007 [99]  moderate | Diabetes type 2 | Counseling or self-directed guide (provider, print material) | Activate (psychological strategies) | | + |
| Sigurdardottir 2007 [100]  moderate | Diabetes type 2 | Education session (provider) | Inform (condition, lifestyle advice), activate (psychological strategies) | | + |
| Blackstock 2007 [101]  moderate | Chronic Obstructive Pulmonary Disease | Education session (provider) | Inform (lifestyle advice), activate (support for condition, monitoring, psychological strategies) | | + |
| Tapp 2007 [102]  high | Acute Asthma | Education session or self-directed guide (provider, print material) | Inform (lifestyle advice), activate (action plans, monitoring, psychological strategies) | | +/- |
| Liddle 2007 [103]  moderate | Low Back Pain | Self-directed guide or counseling (provider, print material, information technology) | Inform (lifestyle advice), activate (support for condition, psychological strategies) | | + |
| Renz 2007 [104]  high | Periodontal Diseases | Education session (provider) | Inform (condition, activities of daily living), activate (psychological strategies) | | + |
| Jackson 2006 [105]  moderate | Diabetes type 2 | Education session (information technology) | Inform (condition), activate (support for condition, monitoring) | | +/- |
| Heneghan 2006 [106]  moderate | Heart disease | Education session (provider) | Activate (action plans, monitoring) | | + |
| Anderson 2005 [107]  low | Depression | Self-directed guide (print material) | Activate (psychological strategies) | | + |
| Deakin 2005 [108]  high | Diabetes type 2 | Education session (provider) | Inform (condition, lifestyle advice), activate (support for condition, action plans, monitoring, psychological strategies) | | + |
| Van Dam 2005 [109]  moderate | Diabetes type 2 | Education session or counseling (provider, lay leader, Internet) | Inform (lifestyle advice), activate (psychological strategies), collaborate (social support) | | +/- |
| Welch 2005 [110]  low | Kidney failure | Education session or counseling or self-directed guide (provider, print material) | Inform (lifestyle advice), activate (support for condition, action plans, monitoring, psychological strategies) | | + |
| Heymans 2005 [111]  moderate | Low Back Pain | Education session (provider) | Inform (condition, lifestyle advice), activate (psychological strategies) | | + |
